# Tetrazine ligation for chemical proteomics

**DOI:** 10.1186/s12953-017-0121-5

**Published:** 2017-06-26

**Authors:** Kyungtae Kang, Jongmin Park, Eunha Kim

**Affiliations:** 1 Department of Applied Chemistry, Kyung Hee University, Yongin, Gyeonggi 17104 Republic of Korea; 2 Center for Systems Biology, Massachusetts General Hospital, Harvard Medical School, 185 Cambridge St, CPZN 5206, Boston, Massachusetts 02114 USA; 3 Department of Molecular Science and Technology, Ajou University, Suwon, 16499 Republic of Korea

**Keywords:** iEDDA reaction, Bioorthogonal reaction, Tetrazine, Chemical biology, Chemical proteomics

## Abstract

Determining small molecule—target protein interaction is essential for the chemical proteomics. One of the most important keys to explore biological system in chemical proteomics field is finding first-class molecular tools. Chemical probes can provide great spatiotemporal control to elucidate biological functions of proteins as well as for interrogating biological pathways. The invention of bioorthogonal chemistry has revolutionized the field of chemical biology by providing superior chemical tools and has been widely used for investigating the dynamics and function of biomolecules in live condition. Among 20 different bioorthogonal reactions, tetrazine ligation has been spotlighted as the most advanced bioorthogonal chemistry because of their extremely faster kinetics and higher specificity than others. Therefore, tetrazine ligation has a tremendous potential to enhance the proteomic research. This review highlights the current status of tetrazine ligation reaction as a molecular tool for the chemical proteomics.

## Background

Chemical proteomics has now become essential for drug discovery and development [1]. Chemical proteomics utilizes chemical probes to understand biological functions of proteins, inform small molecule-protein interactions [2] and validate new druggable protein targets [3]. Compared to techniques in molecular biology and genetics, chemical probes provide powerful tools to perturb protein functions rapidly with temporal and quantitative control [4], enabling good chemical tools to play important roles for chemical proteomics [5]. On the other hand, bioorthogonal chemistry has revolutionized the field of chemical biology by providing powerful chemical tools including metabolite analogous tracking, activity-based protein profiling, target-guided synthesis of enzyme inhibitors, and imaging small molecules in living cells/animals [6–8]. Among bioorthogonal reactions, tetrazine (Tz) ligation has recently emerged as a valuable bioorthogonal coupling tool because of its fast kinetics, spontaneous reactivity without catalysts, and high reaction yield in aqueous solution (and even in serum) [8]. Herein, we described chemical insights of tetrazine ligation and their uses in chemical proteomics.

## Bioorthogonal cycloaddition reactions

**Fig. 1:**
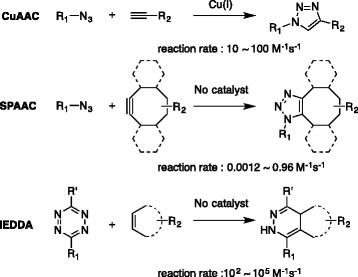
Bioorthogonal cycloaddition reactions including copper-catalyzed azide-alkyne Huisgen 1,3-dipolar cycloaddition (CuAAC), strain promoted copper-free azide-alkyne [3 + 2] cycloaddition (SPAAC) and inverse electron demand Diels-Alder cycloaddition (iEDDA)

Among 20 different bioorthogonal reactions [9]—reactions that do not interfere with biological process [10]—there has been particular progression in cycloaddition reactions (Fig. 1). Beginning from its first introduction by Sharpless et al. in 2001 [11], the concept of “click chemistry” has attracted tremendous interests in the scientific community especially for biomolecule labeling. The initiation was copper-catalyzed azide-alkyne Huisgen 1,3-dipolar cycloaddition (CuAAC) [12, 13]. CuAAC reaction is based on [3 + 2] reaction of azide with terminal alkyne, catalyzed by Cu(I) salt. [14, 15]. CuAAC reaction has reaction rate of 10^1^ ~ 10^2^ M^−1^ s^−1^, approximately, thus it readily occurs in aqueous condition and forms stable triazole as a product [15]. Although CuAAC has been widely used for biomolecule labeling, it is often limited to specific conditions or experiments because of Cu(I) metal catalyst. Therefore there was a high demand for bioorthogonal cycloaddition reaction without metal catalysts to overcome the limitations. A noteworthy progression in this field was the strain promoted copper-free azide–alkyne [3 + 2] cycloaddition (SPAAC) chemistry by Bertozzi and co-workers, which allowed the use of the bioorthogonal cycloaddition reaction in living systems [16]. Introduction of ring strain into the alkyne facilitates the cycloaddition reaction without Cu(I) metal catalyst still with comparable reaction rate (10^−2^ to 1 M^−1^s^−1^) to CuAAC [17]. After the discovery, SPAAC has been significantly used to study proteins and biomolecules in live cells, and even in living organisms [7, 17–19]. More recently, the tetrazine-strained alkene [4 + 2] inverse electron demand Diels–Alder cycloaddition (iEDDA) was introduced for bioorthogonal applications [11]. iEDDA has a tremendously faster reaction rate than SPAAC. The reaction between *trans*-cyclooctene (TCO) with tetrazines showed a reaction rate up to 10^5^ M^−1^s^−1^ [9]. After initial inspiring applications, remarkable applications were published especially in the fields of life sciences. Thanks to its high selectivity, fast reaction kinetics, and non-catalytic nature, the iEDDA cycloaddition reaction has emerged as a state-of-the-art approach for selective bioconjugation in live cells and became an inevitable molecular tool for chemical biologists [9, 12, 20, 21].

## Tetrazine and [4 + 2] cycloaddition

**Fig. 2:**
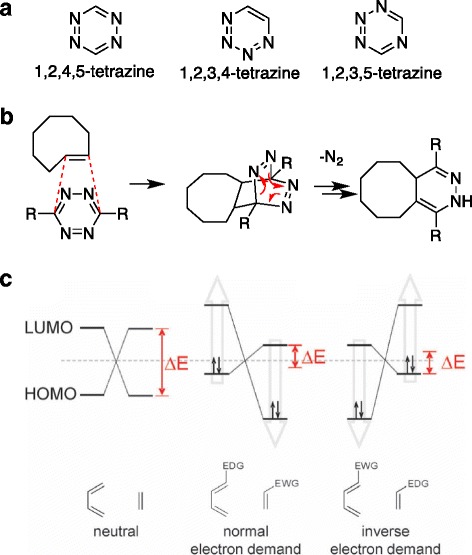
**a** Three different possible tetrazine isomers. **b** schematic illustration of mechanism of iEDDA reaction. **c** frontier orbital model of neutral, normal electron demand Diels-Alder reaction and iEDDA reaction. Reprinted with permission from ref 38. Copyright 2008 American Chemical Society

Tetrazine, voracious dienes for the iEDDA reaction, consists of a six-membered aromatic ring containing four nitrogen atoms (Fig. 2a) [21, 22]. Among three different possible tetrazine isomers, 1,2,4,5-tetrazine is used for the iEDDA reaction [23]. Tetrazine ligation reaction is referred to as the Carboni-Linsey reaction [24], and the completion of the reaction releases N_2_ gas as the only byproduct, which makes the iEDDA reaction irreversible and more suitable for bio-labeling than conventional reversible Diels-Alder reactions (Fig. 2b). Sauer et al found [4 + 2] cycloaddition of tetrazine undergoes in the iEDDA fashion and therefore electron deficient tetrazine took part in LUMO_diene_ and dienophile took part in HOMO_phil_ of the reaction (Fig. 2c). Consequently, electron withdrawing substitution on 3- and 6- position of the tetrazine lowered the LUMO of the diene and therefore accelerate the reaction [20, 21]. Recently, the iEDDA reaction has been redirected as an attractive bioorthogonal decaging reaction [25–27]. Interestingly, both electron donating group (EDG) and electron withdrawing group (EWG) decreased the decaging process. For example, Peng Chen group systematically studied the kinetic effect of substituents on tetrazine for decaging reaction [27]. They synthesized symmetric tetrazine having the same substituents on 3- and 6- position of tetrazine. They found that the substitution of an EDG on tetrazine hindered the decaging process due to an increased LUMO energy level. The decaging process with tetrazine/TCO chemistry consists with an initial iEDDA reaction step followed by a subsequent elimination step. Therefore, an increased LUMO energy level decreases the reaction rate of the conjugation step for the decaging process. On the other hand, they found that the substitution of an EWG group on tetrazine suppressed the following elimination step. Finally, they found that unsymmetric tetrazine having an EWG and a small alkyl group on 3- and 6- position enhanced the decaging activities significantly, compared to the symmetric tetrazine.

## Tetrazine-Fluorophore

**Fig. 3:**
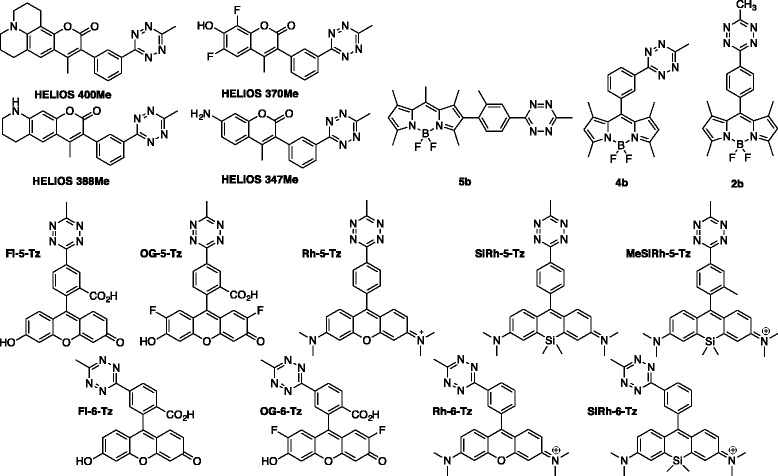
Chemical structures of fluorogenic tetrazine fluorophores

One of the interesting features of tetrazine in terms of imaging is the fluorescence quenching effect of tetrazine. In other words, tetrazine moiety serves as a reactive group for the iEDDA reaction and a fluorescence quencher at the same time. Therefore, tetrazine fluorophores can generally serve as a fluorogenic probe during the iEDDA reaction (Fig. 3). The first discovery of the effect was reported by the Weissleder group [28]. They found that simple conjugation of tetrazine to fluorophores generally reduced the fluorescence intensity of the fluorophore. Interestingly, after the iEDDA reaction, they found that the fluorescence intensity of the fluorophore had recovered. Based on that the maximum quenching effect was observed with BODIPY-tetrazine fluorophore, they concluded that the quenching effect was due to energy transfer from fluorophore to the tetrazine moiety [8]. Soon after they reported newly designed tetrazine fluorophores containing BODIPY and coumarin moieties with thousand to ten thousand folds enhanced fluorescence efficiency after the iEDDA reaction [29, 30]. Recently, fluorogenic tetrazine probes having a more bathochromic shifted emission wavelength were reported from the Wombacher group [31], allowing the iEDDA reaction with fluorogenic tetrazine fluorophores to cover the full visible range of wavelength (Table 1).

**Table 1:** Photophysical properties of fluorogenic tetrazine fluorophores

probe	Ex/Em (nm)	ε (M^−1^cm^−1^)	Φ	Fluorescence Enhancement
HELIOS347	347/455	18500	0.29	2500
HELIOS370	370/463	19000	0.49	2900
HELIOS380	388/482	20000	0.38	11000
HELIOS400	400/502	16000	0.41	4000
2b	NA	NA	0.80	900
4b	NA	NA	0.73	1600
5b	NA	NA	ND	ND
Fl-5-Tz	495/521	57000	0.0037	72
Fl-6-Tz	495/517	55000	0.0033	109
OG-5-Tz	495/524	87000	0.0048	60
OG-6-Tz	495/522	70000	0.0041	103
Rh-5-Tz	556/580	46000	0.037	22
Rh-6-Tz	554/577	54000	0.031	12
SiRh-5-Tz	650/665	58000	0.020	1.8
SiRh-6-Tz	649/664	62000	0.017	3.7
MeSiRh-5-Tz	651/666	53000	0.026	2.0

## Tetrazine ligation reaction in protein imaging

Fluorescence imaging has enabled the non-invasive visualization of the innate functions of biomolecules to understand their functions in biological systems [32]. In this context, discovery of green fluorescent protein revolutionized the many areas of biology [33]. Remarkable advances in fluorescence imaging techniques allowed it playing important roles not only in basic sciences but also in clinical applications [34]. Therefore, using chemical tools for fluorescence imaging is becoming inevitable for the cutting edge chemical proteomics [35]. Initial demonstrations of tetrazine ligation as a bioconjugation method for fluorescent imaging of protein were independently reported from two different research groups in 2008 [36, 37]. For example, the Fox group first demonstrated an iEDDA reaction between TCO and dipyridal tetrazine in organic solvents, water, standard cell nutrient media, or even in cell lysate [36]. They found the second-order rate constant for the reaction to be 2000 (±400) M^−1^s^−1^ in 9:1 methanol/water mixture. They also confirmed that TCO modified thioredoxin can be successfully labeled with tetrazine. Soon after, the Weissleder group utilized tetrazine-dienophile reaction for live-cell protein imaging [37]. After modification of trastuzumab with TCO, they treated the modified trastuzumab to Her2/neu overexpressing SKBR3 cells and then visualized by tetrazine-VT680.

**Scheme 1:**
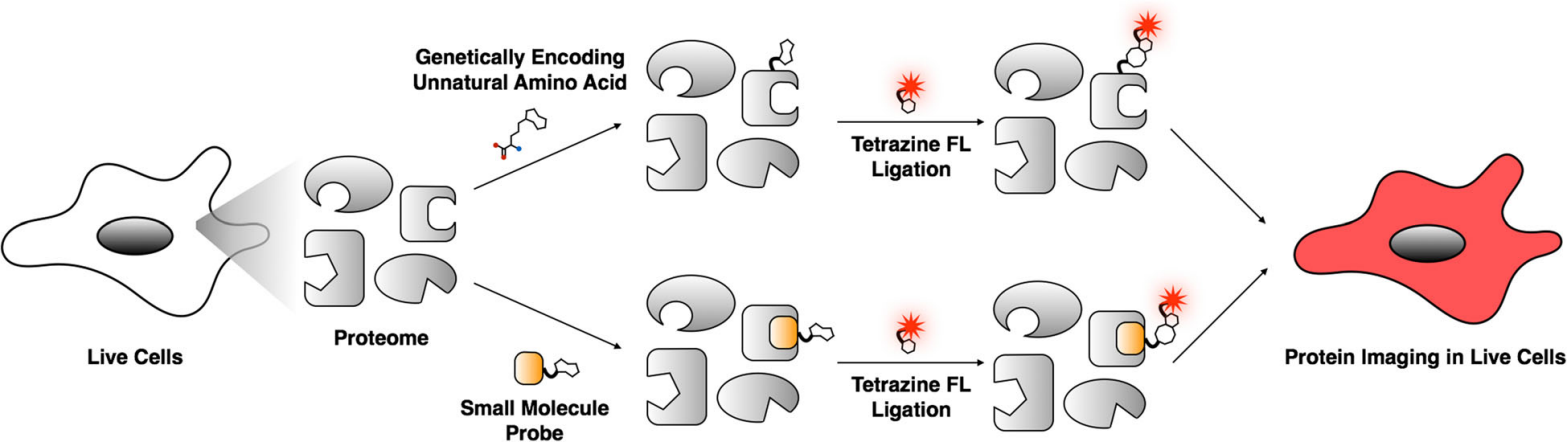
Protein imaging using tetrazine ligation. Top flow : A protein of interest is genetically incorporated with bioorthogonal group embedded unnatural amino acid (UAA). Consequently, UAA is conjugated to tetrazine fluorophore (FL). Bottom flow : A small molecule probe having bioorthogonal group is incubated with proteome and binds to a protein of interest. The probe is conjugated to tetrazine FL for visualization

**Fig. 4:**
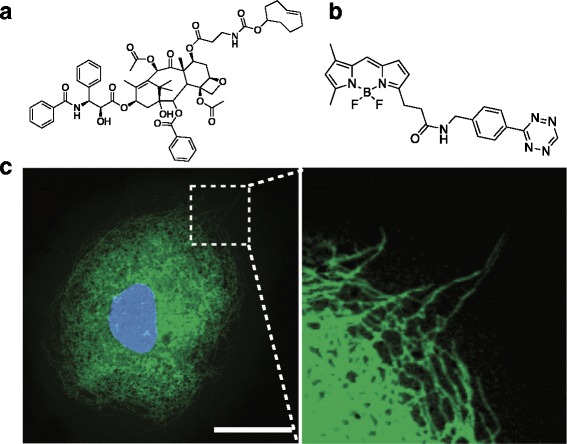
Chemical structure of *trans*-cyclooctene-taxol (**a**) and tetrazine-BODIPY FL (**b**). Confocal microscopy of a kangaroo rat kidney cell after treatment with *trans*-cyclooctene-taxol followed by tetrazine- BODIPY FL (*green*). The nucleus is visualized using Hoechst stain (*blue*). Scale bar: 30 μm. Expansion of the section indicated by the *dashed white line*. Reprinted with permission from ref 29. Copyright 2010 John Wiley & Sons, Inc.

**Fig. 5:**
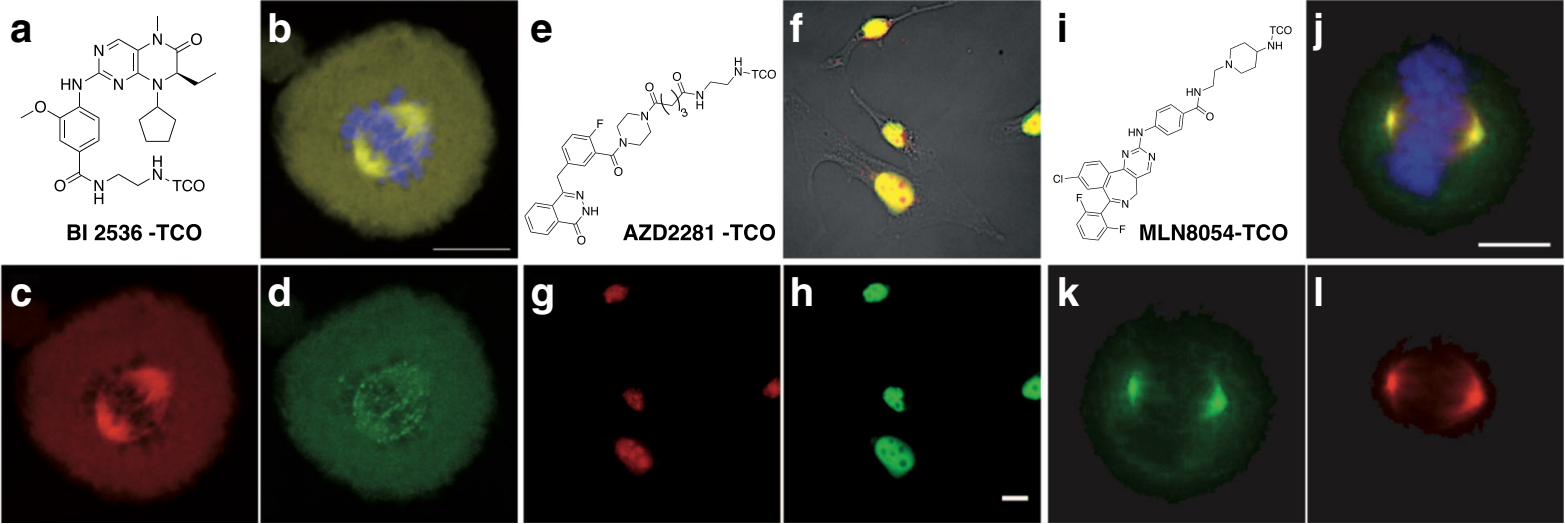
Protein imaging with tetrazine ligation using small molecule-TCO conjugates. **a**, **e** and **i** chemical structure of BI 2536-TCO, AZD2281-TCO and MLN8054-TCO, respectively. **b**, **f** and **j** Merged image of **c & d**, **g** & **h**, and **k** & **l**, respectively. scale bar: 10 μm. **c** BI 2536-TCO/Texas Red-Tz staining (**d**) GFP-PLK1 in PANC-1 cells. **g** AZD2281-TCO/Texas Red-Tz staining. **h** anti-PARP 1 monoclonal antibody staining. **k** MLN8052-TCO/CFDA-Tz staining. **l** RFP-AKA in PANC-1 cell. (AKA : Aurora Kinase A. CFDA : carboxyfluorescein diacetate. GFP : green fluorescent protein. PARP : Poly(ADP-ribose) polymerase 1. PLK : Polo-like kinase 1. RFP : red fluorescent protein.) Reprinted with permission from ref 40-42. Copyright 2010-2012 John Wiley & Sons, Inc.

Imaging binding partners of a small molecule in live cells was also feasible with the tetrazine ligation reaction (Scheme 1). The first demonstration was labeling TCO-Taxol (Fig. 4a) with tetrazine-BODIPY FL (Fig. 4b) [28]. Based on structure-activity relationship, C7 position of taxol was modified with TCO and kangaroo rat kidney cells were incubated with the TCO-taxol for 1 h. Later, tetrazine-BODIPY FL was treated for 20 min. With this approach, the Weissleder group successfully visualized tubulin protein, a binding partner of taxol compound (Fig. 4c). With this success, various drugs, including Olaparib [38], BI 2536 [39], MLN8052 [40], PF04217903, Foretinib [41] and Dasatinib [42], were modified with TCO for labeling target proteins of the drugs, such as Poly ADP ribose polymerase 1, polo-like kinase 1, aurora kinase A, cMET, ABL1, SRC and CSK (Fig. 5).

Another protein labeling strategy is utilizing unnatural amino acid (UAA) for site-specific modification of protein (Scheme 1). Site-specific protein labeling expanded proteomic research toward mechanistic understanding of protein dynamics, protein-protein interactions, and protein folding. Among bioorthogonal reactions, iEDDA is the most suitable reaction due to its rapid reaction kinetics and metal free reaction mechanism for minimal protein damaging. Fox and Mehl group developed the first UAA, 4-(6-methyl-s-tetrazin-3-yl) aminophenylalanine, for site-specific protein labeling [43]. They evolved the *MjTyrRS*/tRNA_CUA_ pair in *pDule-mtaF* and this allowed for the expression of a UAA containing GFP in response to the Amber codon. Because of the quenching property of tetrazine for GFP fluorescence signal, they could measure the reaction rate of 4-(6-methyl-s-tetrazin-3-yl) aminophenylalanine incorporated GFP with *s*-TCO by measuring increase of the fluorescent signal, and the confirmed reaction rate was faster considerably than other site-specific labeling both in vitro and in *E. coli* (880 and 330 M^−1^ s^−1^, respectively). Soon after the first demonstration of site-specific cellular protein labeling via the iEDDA reaction, strained alkene and alkyne containing UAAs (including Norbornene [44–47], bicyclo [6.1.0]-nonynes [46], *trans*-cyclooctenes [46, 47] and 1,3-disubstituted cyclopropenes [48]) have been successfully incorporated site-specifically into proteins expressed in *E. coli* and mammalian cells by engineering tRNA_CUA or_ tRNA^Pyl^/PylRS pairs [49] (Table 2). Starting from the GFP modification, enthusiastic endeavors allowed to incorporate the bioorthogonal UAAs not only into cell-surface proteins, such as Insulin receptor [47], EGFR [50], and OmpC [51], but also into nuclear proteins, jun [46] and LacI, and into cytosolic proteins, such as actin [52], MEK1/2 [53] and interferon-inducible transmembrane protein 3 [54].

**Table 2:** Unnatural amino acids for iEDDA reaction

UAA functionalities	Reaction partner	Reaction rate (M^−1^ s^−1^)	Condition	Labeled Protein	system	Ref
Tetrazine	*s*-TCO	880 ± 10 (in vitro) 330 ± 20 (in *E. coli*)	*E. coli.*	GFP	tRNA_CUA_/MjTyrRS (Y32E, L65A, A107E, F108P, Q109S, D158G, L162G)	[43]
*trans*-cyclooctene	dipyridyl-Tz	17248 ± 3132 (in vitro)	*E. coli.*, HEK293	sfGFP	tRNA_CUA_/MbPylRS (Y271A, L274M, C313A)	[46]
	Tz-Cy5, Tz-TAMRA	NA 35000 ± 3000	HeLa	GFP	tRNA_Pyl_/PylRS(Y306A, Y384F)	[45]
norbornene	Tz-TAMRA	9	*E. coli.*, HEK293	sfGFP	tRNA_CUA_/PylRS	[44]
	dipyridyl-Tz-DANSYL	NP	*E. coli.*	YFP	tRNA^Pyl^/PylRS(Y394F,Y306G, I405R)	[77]
	Tz-TAMRA	8	*E. coil.*, HeLa	GFP	tRNA_Pyl_/PylRS(Y306A, Y384F)	[45]
BCN	dipyridyl-Tz	1245 ± 45 (in vitro)	*E. coli*., HEK293	sfGFP	tRNA_CUA_/MbPylRS (Y271M, L274G, C313A)	[46]

Although an iEDDA reaction between unstrained olefin and a tetrazine is not kinetically favored, the incorporation of unstrained non-canonical amino acids (NCAAs) was also reported recently. For example, the Liu group surveyed iEDDA reactions between nine different NCAAs and two different tetrazine-fluorescein dyes [55]. After confirming that 10 different unstrained olefins have reasonable reaction kinetics (rate constants range from 1.2 to 81 x 10^−3^ M^−2^ s^−1^) with tetrazine-fluorescein (Table 3), they site-specifically incorporated UAAs to super folder green fluorescent protein (sfGFP), using pyrrolysyl-tRNA synthetase (PylRS) mutant system together with tRNA^Pyl^_CUA_. They confirmed that the incorporated unstrained olefin could be labeled with tetrazine dyes at in vitro condition. Furthermore, they found that an *E. coli* outer membrane protein, OmpX, could be site-specifically labeled with the iEDDA reaction with UAA having unstrained olefin. Recently, the Guo group reported a fluorogenic protein labeling strategy using tetrazine ligation reaction with unstrained alkene [56]. Although styrene-tetrazine reaction (0.078 M^−1^s^−1^) is slower than the reaction between strained alkenes and tetrazine, reaction rate is still comparable with other bioorthogonal reactions and more importantly it can be used as reaction for generating new fluorophore, 4-phenyl-3,6-di(pyridin-2-yl)-1,4-dihydropyridazine (PDHP). Screening of PylRS variants, they found that DizPKRs-Y349F [57] successfully incorporated lysine-derived UAA containing styrene moiety (KStyr) into Asn149 position of sfGFP and Phe 28 position of HdeA protein. Such successful genetic incorporation of KStyr into the proteins allowed them for the fluorogenic labeling of proteins both in vitro and in *E. coli*.

**Table 3:** Second-order reaction rate constant between unstrained olefin dienophiles with Fluorescein-Tetrazine

Entry	Unstrained Olefin	*k* (10^−3^ M^−2^ s^−1^)
1	prop-2-en-1-ol	9.4 ± 1.2
2	(*E*)-but-2-en-1-ol	1.9 ± 0.1
3	but-3-en-1-ol	26 ± 5
4	pent-4-en-1-ol	36 ± 4
5	2-(vinyloxy) ethan-1-ol	81 ± 1
6	(*R*)-*N*-(5-aminohexyl) acrylamide	1.2 ± 0.1
7	(*R*)-*N*-(5-aminohexyl) but-3-enamide	1.7 ± 0.2
8	(*R*)-*N*-(5-aminohexyl) pent-4-enamide	11 ± 2
9	(*R*)-*N*-(5-aminohexyl) hex-5-enamide	16 ± 1
10	(*R*,*E*)-*N*-(5-aminohexyl)-3-methoxyacrylamide	19 ± 1

Fluorescein-Tetrazine *= N*-(2-(3-(3′,6′-dihydroxy-3-oxo-3*H*-spiro[isobenzofuran-1,9′-xanthen]-5-yl) thioureido) ethyl)-4-(1,2,4,5-tetrazin-3-yl) benzamide

## Comparison of bioorthogonal click reactions in target identification

**Scheme 2:**
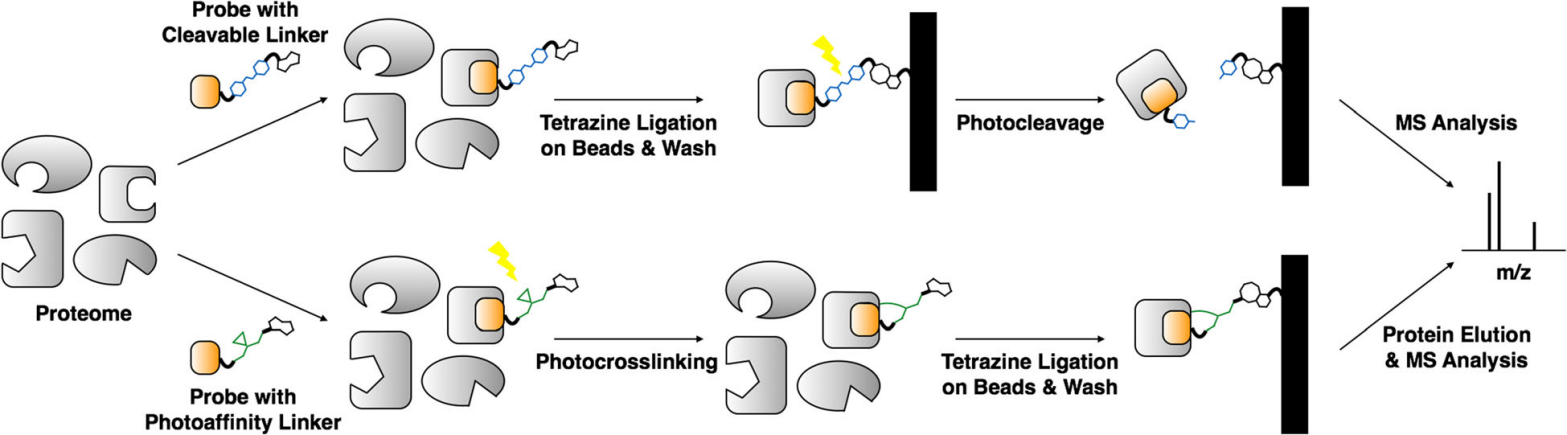
Target Identification (ID) using tetrazine ligation. Top flow : Target ID probe with photocleavable linker is incubated with proteome and binds to target protein. The target protein is conjugated to beads with tetrazine ligation for purification. The bound proteins are photo-cleaved from the beads and analyzed by LC-MS/MS. Bottom flow : Target ID probe with photoaffinity linker is incubated with proteome and binds to target protein. UV irradiation generates a covalent bond between target protein and the probe. The proteins are conjugated to beads with tetrazine ligation for purification. The bound proteins are denatured and eluted from the beads, followed by MS analysis

**Fig. 6:**
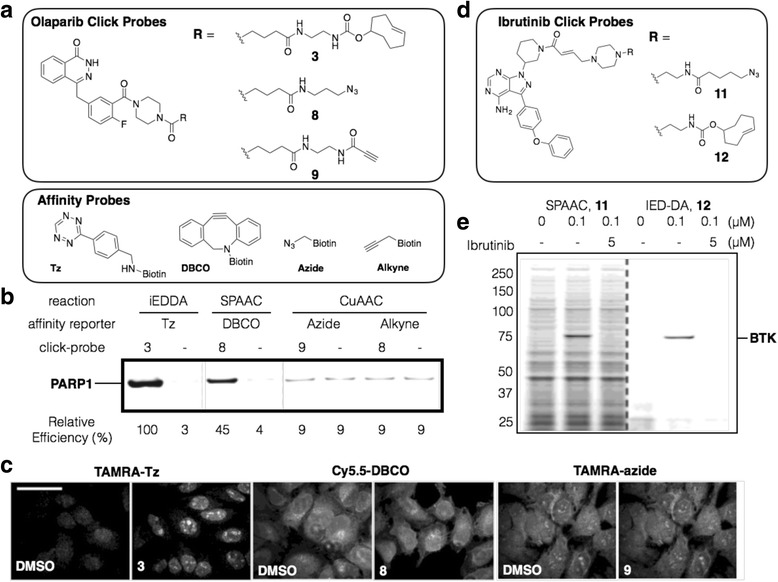
Target identification of Olaparib and Ibrutinib. **a** Structure of Olaparib target ID probe with diverse bioorthogonal groups. **b** Comparison of reaction efficiency of iEDDA, SPAAC, and CuAAC in affinity based enrichment (pull-down) assay. HuT78 nuclear lysate was incubates with target ID probes (**3**, **8**, **9**) and conjugated with biotin for 30 min (probe **3**), 45 min (probe **8**) and 90 min (probe **8** and **9**). THPTA and sodium ascorbate were used for a ligand and a reducing agent of CuAAC. The target proteins were enriched using neutravidin beads, released from the beads and immunostained with anti-PARP1 antibody. The target protein enrichment efficiency was calculated based on quantification of PARP1 bands. **c** Comparison of reaction efficiency of iEDDA, SPAAC, and CuAAC in fluorescent cell imaging. 2 μM of target ID probes **3**, **8**, **9** or DMSO were treated to HeLa cells for 1 h, followed by fixation and permeablized. **3**, **8**, **9** were then conjugated with 100 nM TAMRA-Tz for 5 min, 10 μM Cy5.5-DBCO for 60 min and 10 μM TAMRA-azide for 60 min, respectively (scale bar: 25 μm). **d** Structure of Ibrutinib target ID probe functionalized with azide and TCO. **e** Comparison of target protein (BTK) specificity of SPAAC and iEDDA in fluorescent gel imaging. BV-173 lysate was treated with **11** and **12** in the presence or absence of Ibrutinib. Protein binding target ID probes were labeled with Cy5.5-DBCO or Cy5-Tz. Then the labeled proteins were visualized by SDS gel electrophoresis and fluorescence gel scanning. Reprinted with permission from ref 63. Copyright 2016 American Chemical Society

Since Cravatt et al. reported alkyne-azide cycloaddition (CuAAC) click reaction for labeling proteins of interest in whole cell proteome [58], CuAAC has been used to explore biological system in broad spectrum of researches [59]. Despite its huge potential in biological applications, copper mediated protein degradation, long reaction time and low reaction yield in aqueous solution were a big huddle in proteomic research [7]. Bertozzi and Weissleder group have reported copper-free SPAAC [16] and iEDDA [37] as new bioorthogonal click reactions for biological research. With increased reaction yield and fast reaction time, SPAAC and iEDDA improved fluorescent cell imaging and protein labeling. Successful protein imaging of bioorthogonal click chemistry led its application toward small molecule target protein identification (target ID). Instead of fluorescent dye, biotin linkers are conjugated to proteome-labeling target ID probes through the click reaction. Then, the target proteins are isolated using streptavidin beads and identified with LC-MS/MS analysis (Scheme 2). In contrast to CuAAC, no-copper mediated protein degradation and high reaction yield of SPAAC and iEDDA were expected to bring increment of the target protein enrichment yield. Rutkowska et al. recently reported comparison of different bioorthogonal click chemistries for target ID [60]. PARP targeting Olaparib was conjugated with alkyne, azide or TCO for three different click reactions, CuAAC, SPAAC, and iEDDA; **3**, **8**, and **9** respectively (Fig. 6a). Each target ID probes (**3**, **8**, and **9**) were incubated with cell lysates for target protein binding and conjugated with tetrazine (Tz) -biotin (iEDDA), DBCO-biotin (SPAAC), azide-biotin or alkyne-biotin (CuAAC). Target proteins bound to probes were enriched with neutravidin beads, thereby isolated from rest of the proteins (Pull-down assay). The isolated proteins were then released from the beads and were visualized by western blot (Fig. 6b). It is noteworthy PARP1 enrichment efficiency using iEDDA was 100%, but SPAAC and CuAAC gave only 45 and 9% efficiency, respectively. Therefore, iEDDA is not only the fastest reaction among three different click reactions but also gives high reaction yield for target protein enrichment. In cellular fluorescence imaging, Cy5.5-DBCO and TAMRA-azide exhibited high background signals, but TAMRA-Tz did not (Fig. 6c). These results indicated that iEDDA has high reaction efficiency and specificity for target protein labeling. This finding was also observed in target ID for Ibrutinib. First of all, Ibrutinib was conjugated with azide (**11)** or TCO (**12)** for target ID probe synthesis. **11** or **12** were incubated with proteome, resulting mixture was incubated with DBCO-Cy5 or Tz-Cy5, respectively, and the labeled proteome was run on SDS gel electrophoresis and visualized with in-gel fluorescence scanning. Interestingly, strong background protein labeling was observed with **11** (SPAAC reaction), however, **12** (iEDDA reaction) stained target protein of Ibrutinib, Brutons Tyrosine Kinase, very specifically and barely labeled non-target proteins.

## Cellular target protein occupancy assay

**Fig. 7:**
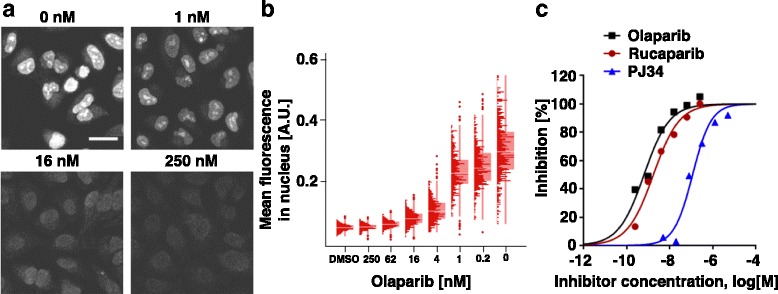
Target protein occupancy measurement for PARP1 inhibitors. HeLa cells were treated with DMSO or Olaparib target ID probe **3** in the presence of indicating concentration of Olaparib. The cells were fixed and permeablized and protein binding **3** were conjugated with 100 nM Cy5-Tz for 5 min (scale bar: 50 μm). **b** Mean fluorescence of single cell nuclei (*n* = 300—520) with different concentration of Olaparib was shown in *box plot*. Histograms superimposed on the *box plot* was distribution of individual fluorescence intensities. Plotted data was from one representative experiment of (**a**). **c** PARP1 occupancy assay of Olaparib (*black squares*), Rucaparib (*Red circles*), and PJ34 (*Blue triangles*). Fluorescence intensity of **3** inside nuclei was measured in the presence of each compound. Reprinted with permission from ref 63. Copyright 2016 American Chemical Society

Target ID probes bind to target protein in live cell and give information about target protein location and expression level inside the cells [61]. Excess amounts of drugs and target ID probes will compete each other to bind target proteins and fluorescent signal quantification of target ID probe inside cells will provide target protein occupancy of drugs. EC_50_ value of the drug can be determined from residual fluorescence signal of target ID probes. This observation could give drug binding information even in a single cell level for therapeutic researches. Rutkowska et al. used Olaparib target ID probe (Olaparib-TCO, **3**) to measure Olaparib target protein engagement (Fig. 7a) [60]. With fixed concentration of **3** (1 μM), increasing Olaparib concentration reduced the cellular fluorescence intensity. Using confocal fluorescence microscope, fluorescence intensity of several hundred nuclei were quantified; cellular PARP1 pEC_50_ for Olaparib was 9.2 (Fig. 7b). Then, target ID probe **3** was also used for pEC_50_ measurement for structurally distinct PARP1 targeting compounds Rucaparib and PJ34 (Fig. 7c). This data implicated that target protein occupancy assay can not only measure the binding affinity of drugs but also rank the affinity of small molecules targeting same protein. Further optimization of this assay could be a useful strategy to understand drug pharmacokinetics in cells and even in vivo studies [62].

## Cleavable linker in target ID

**Fig. 8:**
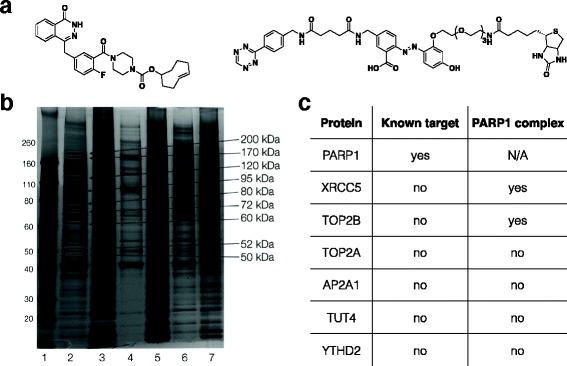
Olaparib target ID with a cleavable linker. **a** Structure of Olaparib-TCO and a tetrazine-biotin cleavable linker. **b** Pull-down assay for Olaparib-TCO binding proteins. A2780 proteome were incubated with Olaparib-TCO and conjugated with Tz-biotin cleavable linker. After target protein enrichment using streptavidin beads, bead binding proteins were eluted by adding dithionite (DT). Eluted proteins were resolved in SDS gel electrophoresis and silver-stained. Lane 1 : marker, Lane 2 : Olaparib-TCO, cleaved with 25 mM DT, Lane 3 : protein left in beads from lane 2, Lane 4 : Olaparib-TCO, cleaved without DT, Lane 5 : protein left in beads from lane 4, Lane 6 : DMSO, cleaved with 25 mM DT, Lane 7 : protein left in beads from lane 6. Protein sizes on the right indicate bands for LC/MS-MS protein analysis. c List of identified target proteins. Beyond target protein PARP1, unknown target protein candidates are discovered. Reprinted with permission from ref 69. Copyright 2013 John Wiley & Sons, Inc.

In general target ID process, bioactive small molecules are covalently attached to biotin linkers and immobilized on streptavidin-coated beads. Target proteins of small molecule bound to the beads are isolated from cell lysates through intensive washing steps. Isolated target proteins are released from the beads through either by trypsinization or streptavidin denaturation [63]. Aside from the protein of interest, non-specific binding of other proteins to the beads could be mixed with real binder of the bioactive compound, which often gives false positives for target identification. To address this issues, diverse biotin linkers have been developed [64, 65]. One example is a cleavable linker for effective release of small molecule binding proteins from beads (Scheme 2). For example, phenylazobenzoic acid moiety could be cleaved in 20 second by reacting with sodium dithionite (Na_2_S_2_O_4_). Yang et al. used this moiety to synthesize a new biotin linker for Olaparib target protein enrichment [66]. First of all, target ID probe for Olaparib was synthesized by conjugating Olaparib to TCO. A cleavable linker for the probe was synthesized by conjugating tetrazine to biotin with phenylazobenzoic moiety in between (Fig. 8a). MHH-ES1 Ewing’s sarcoma cells and A2780 ovarian cancer cells were treated with Olaparib-TCO, and the cells were washed with media to remove excess Olaparib-TCO. The cells were lysed and resulting lysates were incubated with streptavidin magnetic beads, pre-labeled with Tz-phenylazobenzoic acid-biotin linkers, for target protein enrichment. After intensive washing to remove unbound proteins, the linker was cleaved by treatment of sodium dithionite (DT) and thereby only small molecule bound proteins were released from the beads, leaving nonspecific binding proteins remained on the beads. They also collect nonspecific protein cleavage from the beads by replacing DT with buffer only. Released proteins were separated by SDS-PAGE, visualized by silver staining (Fig. 8b) and protein bands from DT treatment were excised and trypsinized for LC-MS analysis. Beyond a classical known target protein of Olaparib, PARP1, unknown Olaparib-binding proteins were identified, which was shrouded by nonspecific bead binding proteins in conventional pull down methods (Fig. 8c). This result implicates importance of linker design and type of bioorthogonal chemistry in target ID. Combination of tetrazine ligation and cleavable linker design strategy showed a new area in target ID.

## Photoaffinity based target ID probe

**Fig. 9:**
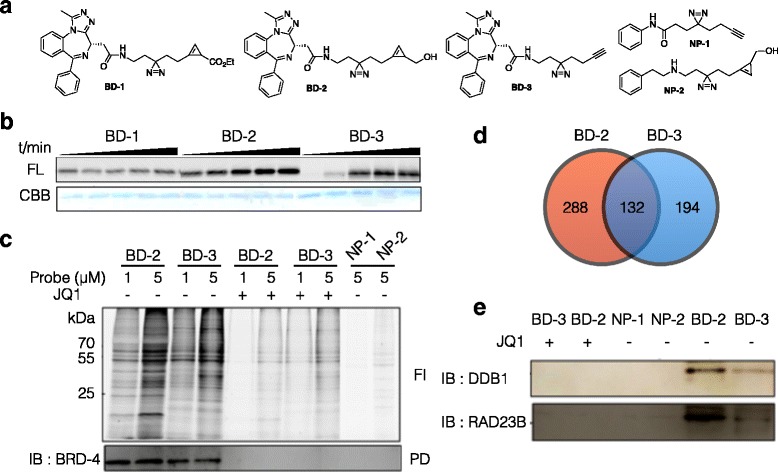
Photoaffinity based target ID of bromodomain inhibitor (+)-JQ1. **a** Structure of (+)-JQ1 target ID probe containing diazirine and cyclopropene. NP-1 and 2 are negative probes for control. **b** Time dependent target ID probe labeled BRD-4 conjugated with TER-Tz (for **BD-1** and **2**, iEDDA) or TER-azide (for **BD-3**, CuAAC). FL : in-gel fluorescence scanning. CBB : Coomassie staining. TER : Tetraethyl Rhodamine. **c** Proteome labeling of live HepG2 cells by **BD-2** or **BD-3** with or without excess amount of (+)-JQ1. The pull down and immunostaining showed BRD-4 enrichment by target ID probes. **d** Ven diagram showing number of **BD-2** or **BD-3** (1 μM) binding proteins. **e** Target protein validation of newly discovered unknown target proteins. HepG2 proteome was labeled by 1uM **BD-2**/**3**. Negative probes and **BD-2**/**3** with excess amount of (+)-JQ1 did not label target proteins. Reprinted with permission from ref 76. Copyright 2014 American Chemical Society

Affinity-based pull down methods had been considered as a gold standard method in target ID. The biggest limitation of this approach is that non-covalent small molecule-target protein interaction is dependent on experimental conditions such as buffers, temperature, incubation time, and washing conditions [67]. Photoaffinity based target ID overcomes those limitations by UV induced covalent bond generation between small molecule and interacting proteins [68]. The covalent bonding secure small molecule-protein interaction in various experimental conditions [69, 70]. Moreover, weak binding or low abundant target proteins can be tractable from huge amount of other non-target proteins in cell lysate [71]. In photoaffinity based target ID, alkyne has been mainly used as a bioorthogonal functional group for CuAAC [72]. Recently, Yao et al. used iEDDA for target ID probe design and identified unknown target proteins of, Bromodomain (e.g. BRD4) inhibitor, (+)-JQ1 (Fig. 9a) [73]. Instead of TCO, smaller size cyclopropene was used as a dienophile for minimal target ID probe design in this research. For the comparison, two types of cyclopropene and alkyne containing diazirine photoaffinity linkers were synthesized and conjugated to (+)-JQ1 to generate target ID probe **BD-1**, **-2**, and **-3. NP-1** and **2**, photoaffinity linker with only benzene group, were also synthesized as negative control probe. To test BRD4 labeling efficiency, the probes were incubated with recombinant BRD4 and were covalently conjugated to target protein by following UV irradiation. Resulting lysates were then labeled with tetraethyl-rhodamine-tetrazine (TER-Tz) or tetraethyl-rhodamine-azide (TER-N_3_) and visualized by fluorescence gel scanning. Time dependent target protein labeling efficiency of each probes were evaluated and showed that **BD-2** was the best probe (Fig. 9b). In proteome profiling in HepG2 cells, **BD-2** and **3** proteome labeling gave potential target protein candidate bands in gel. As in recombinant BRD-4 labeling, **BD-2** showed higher proteome labeling efficiency compared to **BD-3** (Fig. 9c). Cellular proteome labeling, and target protein binding affinity of **BD-2** was also higher than that of **BD-3**. Negative probes (**NP-1** and **2**) and probes (**BD-2** and **3**) in the presence of 10x (+)-JQ1 barely labeled proteome, demonstrating labeled proteins are (+)-JQ1 target, not nonspecific labeling. LC-MS/MS analysis showed **BD-2** and **BD-3** bind to 420 and 326 proteins, respectively and they share only 132 proteins (Fig. 9d). With Olaparib target ID report [66], **BD-2** demonstrated again the importance of bioorthogonal chemistry in target ID. Among the target protein candidates, DDB1 and RAD23B were selected for further validation. BD-2 and BD-3 labeled proteins were conjugated with biotin, enriched by pull-down and visualized by anti-DDB1 and anti-RAD23B antibodies. Both proteins were identified from **BD-2** and **BD-3** labeled proteome but not with 10x (+)-JQ1, confirming two proteins truly bind to (+)-JQ1 (Fig. 9e).

## Conclusion

Chemical proteomics became one of the most reliable and essential approaches to understand biological phenomenon. One of the most critical issues in chemical proteomics might be finding robust and reliable chemical probes and tools for voyage to explore biological system. Recent remarkable advances in bioorthogonal chemistry for labeling small molecule, protein of interest and biomolecules other than protein, without perturbation of biological system, has been revolutionized the field of chemical biology by providing powerful chemical tools. Among 20 different bioorthogonal reactions, tetrazine ligation has emerged as a most advanced chemical tools because of the fast reaction time, minimal protein degradation, high selectivity and high reaction yield in biological systems for the chemical proteomics. Discovery of tetrazine ligation brought a huge step forward for better understanding of cellular events. Tetrazine ligation enables efficient protein labeling even in live cells and in vivo using small molecules and unnatural amino acid incorporation. It is also used for small molecule target ID with high protein enrichment yield, allowing identification of unknown and low expressed target proteins. This unique bioorthogonal chemistry, tetrazine ligation, is just discovered and explored as chemical tools for the proteomics and, therefore, significant improvements and applications are expected to unveil mysteries of biological systems [74–76].

## Abbreviations

BRD: Bromodomain
CuAAC: Copper-catalysed azide-alkyne Huisgen 1,3-dipolar cycloaddition
DBCO: Dibenzocyclooctyne group
DT: Sodium dithionite
EC_50_: Half maximal effective concentration
EDG: Electron donating group
EWG: Electron withdrawing group
GFP: Green fluorescent protein
HOMO: Highest occupied molecular orbital
iEDDA: Inverse electron demand Diels–Alder cycloaddition
LUMO: Loweset unoccupied molecular orbital
NCAAs: Non-canonical amino acids
PDHP: 4-phenyl-3,6-di(pyridin-2-yl)-1,4-dihydropyridazine
pEC_50_: −Log(EC_50_)
SDS-PAGE: Sodium dodecyl sulfate polyacrylamide gel electrophoresis
sfGFP: Super folder green fluorescent protein
SPAAC: Strain promoted copper-free azide–alkyne [3 + 2] cycloaddition
TAMRA: Tetramethylrhodamine
target ID: Target identification
TCO: *Trans*-cyclooctene
TER-N_3_: Tetraethyl-rhodamine-azide
TER-Tz: Tetraethyl-rhodamine-tetrazine
Tz: Tetrazine
UAA: Unnatural amino acid
